# Colour Cues That Are Not Directly Attached to the Body of Males Do Not Influence the Mate Choice of Zebra Finches

**DOI:** 10.1371/journal.pone.0167674

**Published:** 2016-12-15

**Authors:** E. Tobias Krause

**Affiliations:** 1 Department of Animal Behaviour, Bielefeld University, Konsequenz 45, Bielefeld, Germany; 2 Institute of Animal Welfare and Animal Husbandry, Friedrich-Loeffler-Institut, Dörnbergstr. 25–27, Germany; Claremont Colleges, UNITED STATES

## Abstract

Mate choice decisions of female zebra finches are generally thought to rely on the assessment of male quality, which includes the specific ornamentation of males. A commonly used paradigm to experimentally manipulate a male’s attractiveness is to add a coloured leg ring to the bird. Some studies have shown that female zebra finches prefer or alter their investment in males that have an additional red leg ring compared with males with green leg rings. Whether the coloured artificial ornaments need to be attached to the male’s body or whether environmental colouration could have a similar effect on male attractiveness remains unclear. Here, I investigated this novel context to determine whether female choice between males is affected by environmental colour cues that are not directly attached to the male’s body in four experiments involving 220 zebra finches (*Taeniopygia guttata*).

A first experiment revealed that females chose males with red colour cues in the environmental background over males with green cues in the background. Based on this finding, I conducted follow-up experiments to obtain a deeper understanding of how environmental colour cues affect mate choice. Therefore, I examined whether female choice behaviour or male behaviour was altered in two additional experiments. Both experiments failed to show any effects of environmental colour cues on female choice or on male behaviour. Therefore, I replicated the initial experiment in a fourth experiment. Again replication failed; thus, the initial results indicating that environmental colouration affects mate choice behaviour of female zebra finches were not supported by the three subsequent experiments; thus, the outcome of the first experiment seems to be a false positive. Taking my results together, I found no robust support for the idea that environmental colour cues that are not directly attached to the body of male zebra finches affect female mate choice decisions.

## Background

Zebra finches are highly social Australian songbirds that form lifelong monogamous pairs [1] with low extra-pair paternity in natural settings, i.e., less than 2% of chicks [2]. Both partners, to a certain extent, perform courtship displays, which include courtship dances by both sexes [3,4], songs by the males (reviewed in [5]), and male-specific visual coloured ornaments. These male-specific visual ornaments consist of cheek patches [1,3], flank colouration and breast stripes [1,3]. Furthermore, beak colouration is usually more intense in males than in females and also appears to contributes to male attractiveness (e.g., [6,7]).

Choosing a mate with attributes that signal a high probability of high-quality offspring might be beneficial to females because, under natural conditions, as the mate remains for several breeding attempts [1] and sires the majority of the offspring [2]. Female zebra finches are thought to make their mate choice decision on the basis of these visual and acoustic courtship displays (e.g., [8–11]), while probably also taking olfactory stimuli into account (e.g., [12]).

This role of sexually attractive ornaments on males and a female’s preference for these ornaments have often been utilized to experimentally manipulate mate attractiveness to either obtain a mechanistic understanding of the role of ornaments in mate choice (e.g., [3,13]) or to increase or decrease the attractiveness of individuals (e.g., [14]). For example, male ornaments have been manipulated using dummy birds equipped with different combinations/alternatives of male ornaments [3] to understand the relative importance of different sexual ornamentations. Artificially coloured feathers have been attached to birds [13,15] to study mechanisms of sexual imprinting. Songs have been modified in single traits and tested in playback experiments to determine which motif traits are preferred by females (e.g., [16]).

However, the most common technique for manipulating mate attractiveness has been the addition of coloured leg bands (red = more attractive; green = less attractive). A study in 1981 [17] showed that these bands lead to a shift in the sex ratio, i.e., red-banded males sire more male offspring [17]. Although this study was immediately criticized for several reasons, e.g., aspects of the study design and data analysis [18,19], the paradigm was thereafter repeatedly used in different contexts. The initial sex-ratio effects [17] have been confirmed in other studies (e.g., [20]), whereas other studies failed to support this finding (e.g., [21]) or yielded equivocal results [22].

Other positive effects of leg band colour have been described for the amount of yolk testosterone; increased testosterone supplementation of eggs by females was observed when females paired with a red-ringed male [23]. However, other studies again failed to confirm these effects [21]. In addition, in some studies, red rings have been observed to increase the male courtship rate [24], whereas other studies failed to support this finding [25]. Common preference tests, e.g., choice chambers, also support the positive effects of red rings on mate attractiveness (e.g., [14,26,27]), but again another study failed to support these observations [28].

Thus, the many positive reports on the effects of colour rings on sex ratio, maternal investment, courtship and mate preferences have been countered by contradictory reports. This method of manipulating attractiveness by adding leg rings as artificial ornaments is often considered a useful tool for experimental purposes, despite occasional published studies with contrary findings. Artificial ornaments that are added to the male body are thus tools for manipulating a male’s attractiveness. I make use here of the general idea of colourful artificial ornaments and expand this idea into a novel context. Little is known about whether female choice is also influenced by artificial colour ornaments that are not directly attached to a male’s body but are located in his vicinity in the environment; thus, I tested this novel context in preference tests. There is no evidence that zebra finches employ ornaments not attached to the body. However, other sympatrically occurring Estrildid finches, such as diamond firetails (*Stagonopleura guttata*), make use of items in the environment (i.e., grass stems) by carrying these items in their beaks during courting (e.g., [29]).

Evaluating whether males benefit from courting in a specific environment with a certain colouration that may increase their attractiveness as perceived by females could potentially provide interesting new insights on mate preference and attractiveness in zebra finches and how artificial coloured ornaments might generally increase attractiveness, regardless of whether they are attached to the male body.

Here, I employed preference tests to determine whether such artificial colour ornaments that are not directly attached to the male influence the mate choice of zebra finches. I hypothesized that females would chose males in environments with attractive colour cues (red) more often than males in less attractive environments, with green colour cues.

## Methods

Experiments were conducted at Bielefeld University, Germany with a total of 220 individual zebra finches, which were distributed over 4 experiments. Several weeks before the beginning of each experiment, the birds were transferred from aviaries to cages (83 × 30 cm and 40 cm high), each containing up to four individuals of one sex. Seed food and water were provided *ad libitum*, and germinated seeds plus egg food were provided thrice weekly. During the tests, seeds were provided at a neutral position. All birds had breeding experience and had no prior exposure to the colour stimuli used in the experiments. The birds were ringed with numbered plastic rings (white, grey or black).

### Experiment 1

**Fig 1:**
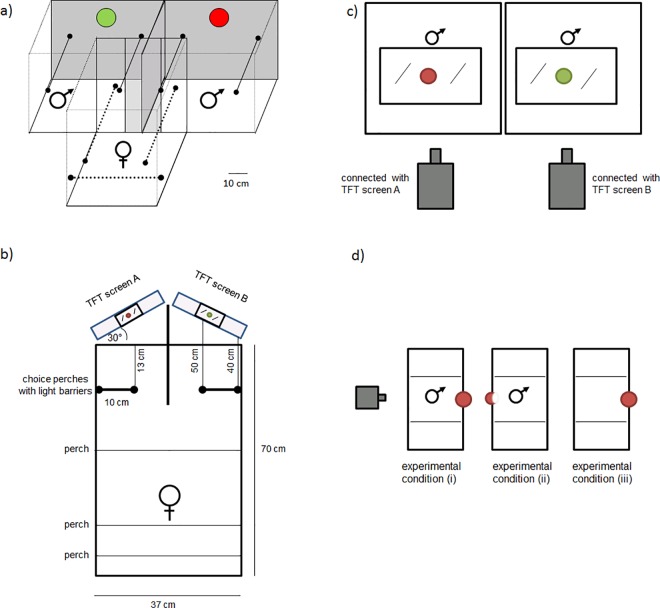
Experimental design used in the experiments: a) Female choice related to males in front of an environmental background with either red or a green colour cues was tested in experiments 1 and 4. The grey-coloured lines in the graphic represent opaque walls. In experiment 2, a video choice set-up was used. b) Females were located in a test cage, which had two perches with light barriers. When sitting at one of these perches, the respective screen became visible to the females, and they could see the males in the cages and the colour spot. c) The male cages were located in an adjacent isolated room, where live video was recorded, and the images were transferred to the female cage. d) Three different experimental conditions regarding the colour spots in the male cages were set up: i) colour spot behind the male, so that it was visible to both the male and female; ii) colour spot in front of the male, so that only the female could see the colour; and iii) colour spot in a cage without a male being present.

Sixty birds (20 females, 40 males) were assigned to twenty distinct trios, each including one female and two males. Females were individually tested for 20 minutes in a dual mate choice set up. In this setup, the female could decide to perch in a neutral zone or on one of two perches that each faced one of the males. Each male was placed in a separate cage with an environmental background that was supplemented with either green or red colour cues (diameter of 10 cm) behind the male (Fig 1A). The males were visually separated from each other. Colony noise was present during testing to reduce the birds’ anxiety and to increase the overall level of activity. Five minutes before the experiment, all of the subjects were placed in the cages; during this time, the female was visually separated from both males. Thereafter, the visual separation was removed, and the first ten minutes of the experiment were performed. Then, the coloured cues in the male cages were exchanged between the two sides, while the males remained. After another five-minute break, the experiment was conducted for a second ten-minute period.

### Experiment 2 –Visual mate choice with different information levels

Sixty zebra finches (20 females, 40 males) were assigned to twenty distinct trios. Each female was subsequently tested under three experimental conditions on one day. The order of the conditions was randomized across the females. Each experimental condition lasted for 2 hours, during which the female could make her decision (Fig 1B) by occupying perches next to the screens. After half of the time period had elapsed, the coloured cues were exchanged between male cages, while the males remained. The experiment included the following three conditions: (i) The colour cues (red and green, diameter 10 cm) were placed behind the male, so that they were visible to both females and males; (ii) the coloured cues (diameter 8.5 cm) were placed in front of the males, and the back of each of the cues was white, so that the colour cues were visible only to the female (in this condition, the diameter of the coloured cues was adjusted as the cues were placed closer to the video camera) (Fig 1D); or (iii) the coloured cues (diameter 10 cm) were placed on the back wall, but no males were present in the cages.

The females were transferred to the choice cage the afternoon before the tests began and were kept there overnight to acclimate to the novel cage. During this phase, the TFT screens were switched off. The choice cage had dimensions of 70 x 37 x 47 cm (Fig 1B). Three neutral perches at different heights were provided at the back of the females’ cages (Fig 1B). A recording of a zebra finch colony was played back during the tests. In the front portion of the cage, in close proximity to the two TFT screens (Eizo Flex Scan HD2442W, 24"), two perches were put in place (10 cm in length, positioned at a height of 35 cm above the ground; Fig 1B), each of which was equipped with a light sensor. These perches represented the choice areas, and the number of hops and the time spent on each perch were recorded via a computer. The front portion of the test cage was covered with an opaque board from floor level up to a height of 30 cm. Only the portion from 30–47 cm high was covered with a transparent grid. The TFT screens were positioned outside the cages at a distance of 50 cm from the perches, at a 30° angle from the respective choice perch (Fig 1B). The two TFT screens were directly connected via HDMI cables to two cameras (Panasonic HDC-SD9) in an adjacent, but acoustically and visually separated room. The cameras projected only the video image and not the vocalisations of the males to the respective TFT screens (Fig 1B and 1C). The cameras were placed 1.0 m away from the male cages. The cages of the two stimulus males (Fig 1D) had dimensions of 30 x 40 x 21 cm. The front grid was replaced with a glass slide to enable an optimal video image. The two male cages were located above each other in two containers that were illuminated (Megaman 20W) and lined with sound-proof material. With exception of the light in the containers, the recording room was completely dark. The males did not see any stimulus females during the recording because the test room was dark, and placing a female in front to the male would have disturbed the live image. The males were also transferred to the experimental room the afternoon before the tests started but were kept in separate cages until the experiments began.

Video setups have been previously used to test mate choice paradigms in zebra finches [30–34], and the use of TFT screens eliminates the image flickering that was a problem with the old cathode ray tube screens [33,35]. In my video setup, a minimum distance of 40 cm between the birds and the TFT screens was employed [36] to enable better control of pixilation and image blurring [35,36].

### Experiment 3—Acoustic performance of males associated with colour cues

For this experiment, I used 20 males and 20 females. I placed one male and one female in two adjacent cages (20 x 40 x 30 cm) separated from each other by an opaque divider for 15 minutes. Thereafter, the divider was removed. Sound was recorded for 25 minutes using a Sennheiser ME66/K6 microphone, which was placed outside the cage and directed towards the male’s cage, and a Marantz PMD 660 recorder. The divider was then reinserted for 15 minutes. Thereafter, a second 25-minute session was recorded. During each trial, the males had coloured cues (diameter 10 cm) at the back of their cage that were either red or green. After the first 25 minutes of the recording, the coloured cues were replaced by cues of the other colour. The colour in the first session was alternated between males. The recordings were analysed using Avisoft 5.2 [37]

### Methods for experiment 4—Replication of the first experiment

I used 20 females and 40 males in this experiment. The testing protocol was identical to that of experiment 1, except that the test cage had slightly different dimensions (125 x 44 x 40 cm).

### Measured parameters and statistical analysis

In experiments 1 and 4, female mate choice was measured over a 20-minute period. At every five-second interval (240 intervals in total), the female’s location was recorded as the perch in front of the males with the red or green colour cues or the neutral perch. The location where the female perched is regarded as a proper indicator for her decision [10]. From the sound recordings in experiment 3, I calculated the song motif rate for the first 5 minutes (i.e., number of motifs in the 5 minutes after the first motif occurred) and the song motif rate over the entire 25 minutes of the recording; both measures were calculated as motifs per minute.

A female decision was categorized as “1” if she spent more than 50% of the intervals that it spent in any of the two choice areas (i.e., excluding the intervals in neutral areas) with the red cue and as “0” if she spent more 50% of the intervals in the choice area with the green cue.

A similar procedure was applied in experiments 2/3, where I determined which background colour cue was associated with a greater time/song motif rate for each male. Female time/male singing performance was categorized as “1” when the time/song motif rates were greater in association with the red colour cues and as “0” when they were greater with the green cues. This categorization was performed to examine what stimulus was chosen at a rate above 50% and, thus, to what stimulus the decision of the focal birds is based on, regardless of the strength of the decision. Here, I was interested in the overall decisions, rather than the strength of the decisions (strengths were additionally captured in experiment 2, see below). I analysed female decisions/male performances using a sign-test with an assumed equal distribution. For the preferences in experiment 1 and 4, an additional one-sample t-test was conducted to examine whether the choices deviated significantly from 0.5.

In experiment 2, I additionally recorded the time [s] the females spent on the choice perches and the number of hops directed towards the perches under every condition. These two parameters allowed me to examine potential difference in the strength of decisions. For both measures, the relative choice of the red-coloured cue [(time/hops at the perch where the red spot was presented)/((time/hops at the perch where the red spot was presented) + (time/hops at the perch where the green dot was presented))] and the sum of the two parameters as a measure of how actively the birds were deciding were calculated. The relative time and number of hops were analysed using linear mixed effect models (LMEs) with individual ID as a random factor; the experimental conditions (three levels: coloured spots in front of, behind or without males) and the order of the experimental conditions (three levels) as explanatory factors; and the female's age and body mass at the time of testing as covariates. The normality of the residual distribution was checked using the Kolmogorov-Smirnov-Lilliefors test. The absolute values of time/hops were compared between the three experimental conditions using Friedman tests, and in the case of a significant finding, pairwise post-hoc comparisons were performed using Wilcoxon tests. All tests were calculated with SPSS 22. The raw data from the experiments are available in S1 Table.

### Ethical note

All birds remained in the laboratory stock after the end of the experiments. The behavioural experiments were performed in accordance with the laws of Germany at that time and did not require specific approval. Housing of the birds was permitted by the local government (Gesundheits-, Veterinär- und Lebensmittelüberwachungsamt, Stadt Bielefeld, Germany; # 530.421630–1, 18/04/2002).

## Results and Discussion

### Experiment 1

**Fig 2:**
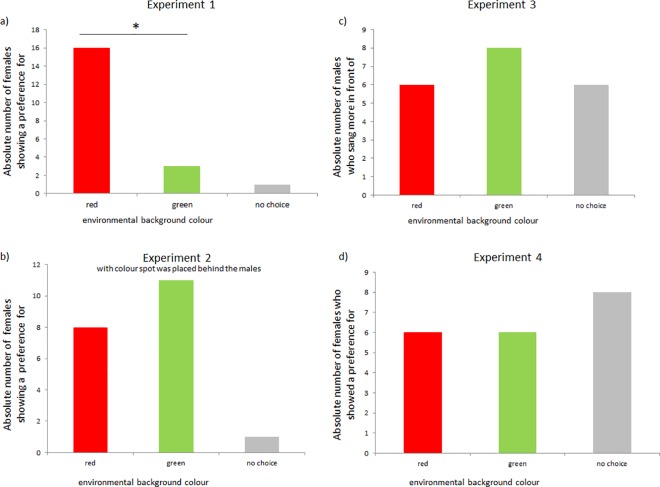
a) Results of experiments: a) Experiment 1: Female choice related to environmental backgrounds with either red- or green-coloured cues in the environment. Females showed a significant preference for the red-coloured cues. b) Results of experiment 2 for the condition where the colour cues were presented behind the males: females showed no preference for the red environmental cues. c) Results of experiment 3: The number of males that sang at higher rates was not different in association with red- versus green-coloured cues. d) Results of experiment 4: in this replication of the first experiment, females showed no preference for the red-coloured cues.

In the first experiment, I addressed the question of whether environmental colour cues affect mate choice decisions of zebra finch females. Females significantly preferred males in front of a red-coloured cue over those in front of a green cue. Among the 20 tested females, 16 were perched at the red cue more often; three at the green cue more often; and one female made no decision (sign test, p = 0.0044; Fig 2A). Although this pattern appeared robust, the preferences were not very strong; i.e., the females spent on average 56% (± 11% S.D.; mean number of intervals for red: 134.4 ± 27.3 S.D.) of the intervals with the red colour cue. Testing the proportion of time spent with red against chance level (0.5) revealed a significant preference for red (one sample t-test, t_19_ = 2.36, p = 0.029). Thus, although the relative preference strength was quite weak compared to other two-way mate choice studies (e.g., 71% in [10]), a systematic significant shift towards red was observed.

However, these results seem to support the idea that female preference for red-coloured ornaments is also generalized to environmental cues, independent of the male making use of such environmental backgrounds. These novel findings could have potentially interesting implications for the understanding of mate choice decisions of zebra finches and may suggest that males might profit from courting in the vicinity of coloured environmental cues. At this stage, whether colour ornaments that are not attached to the males’ body but are present in the near environment a) increase male attractiveness as perceived by females (further examined in experiment 2) and/or b) lead to a change in male behaviour (further examined in experiment 3) remained an open question. With experiments 2 and 3, I aimed to obtain a more detailed understanding of how environmental colour cues affect female mate choice and/or male behaviour.

### Experiment 2 –Visual mate choice with different information levels

In this experiment, I used an automated video-based choice paradigm to exclude any other non-visual cues (i.e., male sounds/odours were excluded). In this test, females could choose between live video images of two males, but the males were unable to view the females. I added two additional experimental conditions to the initial conditions of experiment 1; thus, females were tested in three conditions: (i) The first condition was similar to experiment 1; i.e., a coloured cue was present behind the male, visible to both females and males. In the second condition (ii) the coloured cues were located in front of, rather than behind the males; thus, they were visible only to females. In condition three (iii), only the coloured cues were present (without males) to test whether the females prefer a colour independent of any male presence and whether male presence affects reactivity. These three combinations allowed me to assess whether visual cues affect female preference, male behaviour or both. In contrast to experiment 1, I was unable to replicate that red environmental colour cues increase the proportion of males chosen by females. In none of the three experimental conditions did females show a preference for red-coloured cues. Considering only the absolute choice (red/green), the females showed no significant preference for red when the coloured cues were placed behind the males (8 chose red, 10 green, 1 no choice; sign test, p = 0.65; Fig 2B). The females also did not prefer the red cues when the coloured cues were placed in front of the males, (10 chose red, 9 green, 1 no choice; sign test, p = 1.0). In the condition in which the coloured cues were presented without males being present, the females also did not make any choice (8 chose red, 5 green, 7 no choice; sign test, p = 0.58).

**Fig 3:**
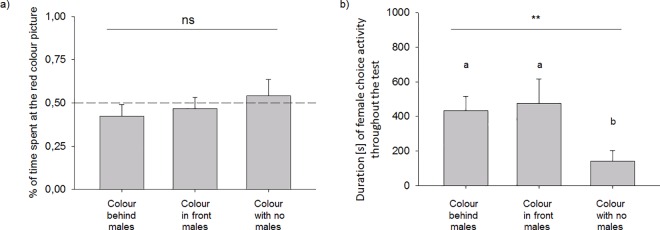
Parameters assessed in experiment 2: a) the proportion of time the females chose the red compartment under the three test settings (not different across conditions); and b) the absolute time they spent choosing under the three test settings (which was significantly different, as indicated by different letters).

Furthermore, a more detailed look in the female decisions revealed that the relative time females spent at the video with the red-coloured cue was not influenced by the three experimental conditions or by any other parameters (LME: factor experimental condition F_2,30.9_ = 0.55, p = 0.59; factor order of experiments F_2,31.1_ = 1.83, p = 0.18; factor female weight at test F_1,13.9_ = 0.80, p = 0.39; factor female age at test F_1,17.1_ = 0.001, p = 0.97; Fig 3A). A similar pattern was found for the number of hops (S1 File). The associations between time and hops and the consistency of the parameters across trials 1 and 2 are shown in the supplement (S1 File).

Interestingly, the absolute time females spent choosing during the tests differed significantly between the three conditions (Friedman test, N = 20, df = 2, Χ^2^ = 16.13, p = 0.0003; Fig 3B). Post-hoc pairwise comparisons revealed that the overall time spent choosing was significantly lower in the condition without males than in the conditions in which the coloured spots were located behind (Wilcoxon test, N = 20, Z = -3.36, p = 0.001; Fig 3B) or in front of the males (Wilcoxon test, N = 20, Z = -3.02, p = 0.003; Fig 3B). The time spent choosing did not differ between the two conditions where males were present (Wilcoxon test, N = 20, Z = -0.26, p = 0.79; Fig 3B). The observation that females spent significantly more time in front of the video images when a male was present than when no male was present indicated that the females perceived the males, which is important to interpret the choice decision in the other contexts, as it indicated that females at least perceived the males in the videos.

Overall, I observed a relatively low strength of female decisions, as throughout the course of the experiment, the females chose one of the two presented males on the choice perches for approximately only 5% of the test duration. This low relative choice time might have been due to the exclusive presentation of visual cues, the relatively long period of testing (6 h), or the lack of responsiveness of the males to the females. The last alternative might have been the case, as the males had no visual contact with any stimulus females due to the experimental settings (see Methods).

Taken together, the results of experiment 2 provide no support for the idea that environmental colour cues that are not directly attached to the male body affect female choice behaviour.

### Experiment 3 –Acoustic performance of males in the presence of colour cues

The male song rate was not affected by the different coloured cues in the environment. The song rate during the first five minutes and during the total duration was higher in six males in the presence of the red-coloured cue. In eight males, the song rate was higher in the presence of the green colour cue (sign test, p = 0.79; Fig 2C). Six birds emitted no songs at all. The average song rate in the first five minutes [throughout the total trial time] was 1.5 motifs/minute ± 2.1 S.D. [0.68 ± 1.1] in association with the red-coloured cues and 1.8 motifs/minute ± 2.2 S.D. [0.79 ± 1.2] in association with the green-coloured cues. Thus, male singing was not affected by the coloured cues in the environment.

Experiments two and three provided no further insight nor support for the idea that environmental colour cues affect female mate choice decisions because they failed to replicate the initial findings. This failure might be due to a lack of biological effect or due to differences in the experimental setting compared with experiment 1. Due to the conflicting results of experiments one versus experiments two and three, I decided to conduct a fourth experiment in which I repeated experiment 1.

### Experiment 4 –Replication of the first experiment

In this replication of experiment 1, females did not prefer males with red-coloured cues in the environment. Among the 20 tested females, 6 preferred the red-coloured cue, 6 the green, and 8 made no decision (sign test, p = 1.0; Fig 2D). Additionally, as in experiment 1, the relative strength of choice for red was weak, i.e., the females spent on average 55% (± 43% S.D.; mean number of intervals for red: 34.5 ± 52.5 S.D.) of the intervals with the red colour cue. When testing this proportion for red against the chance level (0.5), no significant preference was found (one sample t-test, t_12_ = 0.44, p = 0.67). Interestingly, the average relative preference for red was nearly identical to that in experiment 1 (56%), but the standard error in experiment 4 was 4 times higher than that in experiment 1. Thus, experiment 4 also failed to replicate the initial effect. In this experiment, a relatively high proportion of females did not make a choice. However, the proportion of females that chose green was so great that it would not have been possible to observe a significant preference; even if all of the females that did not make a decision had chosen red, the p-value of the sign test would have been 0.12.

When pooling the data from experiments one and four, 22 females preferred males with red-coloured cues, and nine preferred males with green-coloured cues. These pooled data would lead to significance in the sign test (p = 0.031). However, this pooled result is unduly influenced by the female choices in experiment 1, as nearly all of the females made a decision in that experiment. Thus, females from experiment 4 are underrepresented due to the eight females that did not make a decision. The proportion of birds that made a decision differed significantly between experiment 1 (19:1) and experiment 4 (12:8) (Fisher’s exact test, p = 0.02).

## Conclusion

Contrary to the initial hypothesis and the findings of experiment 1, the results for the entire series of four experiments indicate that environmental colour cues that are not directly attached to the male’s body do not have a repeatable, significant effect on the mate choice behaviour of female zebra finches. The initial findings failed to be reproduced in three other experiments. Of course, other biological factors that I did not include here could be responsible for the lack of effect observed in experiments 2, 3 and 4. Although this possibility cannot be fully ruled out, I detected a significant effect in only one of four experiments. Thus, from my perspective, the most likely explanation is that the initial finding was a false positive (type-I error) [38].

Although tests of mate choice in captivity can be only a proxy of natural processes and depend on the applied testing methods [11], they offer the opportunity to manipulate single dimensions/cues within the process of mate choice decision making (e.g., [17,20,27]). Experiments 1, 3 and 4 involved live experiments or stimuli, where the birds had access to multimodal information on the behaviour of a conspecific, whereas in experiment 2, females had access to only visual cues from non-stimulated males. Although these males were probably not courting, the females responded more strongly to them than to video images of empty cages. Considering this particular limitation within experiment 2, the data provide another piece of evidence in line with experiments three and four indicating that environmental colouration does not affect zebra finch mate choice decisions.

Mate choice paradigms appear to be complex with regard to the cues subjects can rely on, and the repeatability of colour ring effects can be problematic, as demonstrated by another study [25]. These authors added colour bands (red/green) to zebra finches and failed to replicate earlier results with regard to courtship rates and body mass; furthermore, they showed in a meta-analysis that overall support from the literature associated with colour bands attached to males in this context is limited [25]. Here, I found similar patterns, but in a new context, where artificial ornaments were not attached to the male body. The effects of artificial colour ornaments on mate choice decisions are not easy to replicate, even within the same laboratory, as shown in the present study. Thus, results regarding the effects of artificial coloured ornaments either attached to the male body (e.g., [17], but see [21]; or [24], but see [25]) or not attached to the male body but present in the environment require validation before making clear conclusions based on these findings.

Taken together, my initial hypothesis that environmental colour cues that are not directly attached to the male body affect female mate choice needs to be rejected on the basis of the series of four experiments.

## Supporting Information

S1 Table Raw data from the four behavioural experiments. Raw data from the four behavioural experiments are presented in these tables
(XLSX)

S1 File Additional results for experiment 2. In this supporting file three additional results for experiment 2 are presented. i) Relative number of hops by the females directed to the red colour cues in the three experimental conditions. ii) Association between the choice parameters: Is the same choice indicated by the relative amount of time and number of hops?. iii) Consistency of female choice between trial 1 and trial 2 in each experimental condition in experiment 2.
(DOCX)
