# Supplementary material for: Colour Cues That Are Not Directly Attached to the Body of Males Do Not Influence the Mate Choice of Zebra Finches
Source: PLoS One. 2016 Dec 15;11(12):e0167674. doi: 10.1371/journal.pone.0167674 (PMC5157975; doi:10.1371/journal.pone.0167674)
Supplement: S1 File — In this supporting file three additional results for experiment 2 are presented. i) Relative number of hops by the females directed to the red colour cues in the three experimental conditions. ii) Association between the choice parameters: Is the same choice indicated by the relative amount of time and number of hops?. iii) Consistency of female choice between trial 1 and trial 2 in each experimental condition in experiment 2. (DOCX) [file pone.0167674.s002.docx]

**S2 File**

**Additional results for Experiment 2**

**Relative number of hops by the females directed to the red colour cues in the three experimental conditions**

The percentage of hops the females directed towards the picture with the red colour cues also was not influenced by the experimental conditions (LME: factor experimental condition F_2,29.58_ = 0.46, p = 0.64; factor order of experiments F_2,29.83_ = 0.66, p = 0.52; factor female weight at test F_1,12.49_ = 0.46, p = 0.51, factor female age at test F_1,15.56_ = 0.24, p = 0.63; Fig. S2a).

The overall number of hops the females made while choosing significantly differed between the three experimental conditions (Friedman test, N = 20, df = 2, Χ^2^ = 7.92, p = 0.019; Fig. S2b). Post-hoc pairwise comparisons revealed that the overall time the females spent actively choosing in the condition without males was significantly lower than in the conditions with males (coloured cues behind the males Wilcoxon test, N = 20, Z = -2.38, p = 0.018; Fig. S2b; coloured cues in front of the males Wilcoxon test, N = 20, Z = -2.17, p = 0.03; Fig. S2b). The total time the females spent choosing did not differ between the two conditions in which males were present (Wilcoxon test, N = 20, Z = -0.69, p = 0.49; Fig. S2b).


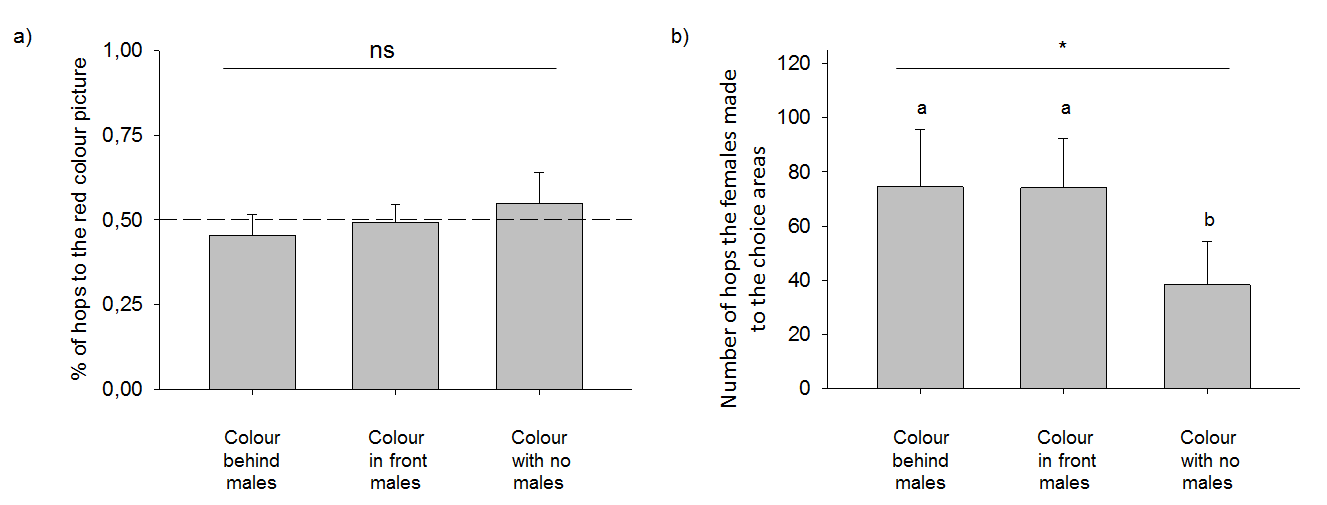


Figure S2: Measures in experiments 2: a) the proportion of hops in the three test setting was not different from each other, but b) the absolute amount of hops was reduced when no male was present.

**Association between the choice parameters: Is the same choice indicated by the relative amount of time and number of hops?**

The choice of the females that was based on the relative amount of time spent at the picture with the red coloured cue was highly correlated with that based on the relative number of hops directed towards the picture with the red coloured cue in all three experimental conditions (coloured cue behind the males N = 19, r_P_ = 0.895; p < 0.0001; coloured cue in front of the males N = 19, r_P_ = 0.93, p < 0.0001; coloured cue presented without males N = 13, r_P_ = 0.906, p = 0.00002). Sample sizes that differ from the initial N = 20 are due to the exclusion of females who did not display a preference in the respective test setting.

**Consistency of female choice between trial 1 and trial 2 in each experimental condition**

The choice of the females in the first trial was negatively correlated with that in the second trial in all three experimental conditions (coloured cues behind the males N = 15, r_P_ = -0.42, p = 0.12; coloured cues in front of the males N = 13, r_P_ = -0.63, p = 0.021, Fig. 6b; coloured cues presented without males N = 7, r_P_ = -0.78, p = 0.038). The low sample sizes in this test is because some individuals only made a choice in one of the two trials.

Samples sizes that differ from the initial N = 20 are due to the exclusion of females who did not make a choice in the respective test setting or that made a choice in only one of the two trials.
